# Perspectives of adults living with HIV attending the opportunistic infections clinic at Chitungwiza central hospital in Zimbabwe towards physical activity: a cross-sectional survey

**DOI:** 10.1186/s13102-023-00676-6

**Published:** 2023-05-01

**Authors:** Farayi Kaseke, Precious R Pamire, Tecla Mlambo, Clement Nhunzvi

**Affiliations:** Faculty of Medicine and Health Sciences, Department of Primary Health Care Sciences, Rehabilitation Sciences Unit, University of Zimbabwe, Harare, Zimbabwe

**Keywords:** Physical activity, Exercise, Physical exercise, HIV/AIDS, Perceived benefits, Perceived barriers

## Abstract

**Background:**

Regular physical activity and exercise have been shown to be of benefit in managing the HIV disease, its complications and the side effects of HAART. The perceptions of those living with HIV toward physical activity and exercises is a key factor in advocating for participation of rehabilitation personnel in the management of this disease. However, this domain remains underexplored in Zimbabwe.

**Methods:**

A descriptive cross-sectional quantitative study was conducted with 327 people living with HIV. Participants were consecutively sampled from Chitungwiza Central Hospital Opportunistic Clinic. The International Physical Activity Questionnaire (IPAQ) and the Exercise Benefits/Barriers Scale (EBBS) were respectively used to measure the physical activity level and perceived benefits/barriers of physical exercise among adults living with HIV. The tools were self-administered. The analyses were done for 323 participants using the Statistical Package for the Social Sciences (SPSS).

**Results:**

The mean age was 41.1 ± 11.0. Females constituted 69.7% (n = 225) of the sample. The majority of participants (n = 184; 57%) described themselves as being highly physically active (3204;IQR = 2139–4441 MET-minutes/week). Most of the participants agreed that physical activity prevented heart attacks with a mean value of 3.34 ± 0.65. The majority perceived psychological outlook as the greatest benefit from physical activity among the benefit sub-scales. Furthermore, most of the participants agreed that it cost too much for them to exercise which had a mean of 3.00 ± 0.88. Family discouragement was the most agreed perceived barrier to physical activity with a mean of 2.91 ± 0.67. Those who perceived physical activity as being beneficial reported less barriers. A statistically significant association was found between level of physical activity and CD4 cell count (*p* = 0.035) as well as with employment status (*p* < 0.05). No statistically significant association was reported between the perceived benefits and the level of physical activity (p = 0.214). A statistically significant association was reported between the perceived barriers and age (*p* < 0.05) as well as with employment status (*p* = 0.006).

**Conclusion:**

Adults living with HIV receiving HAART at Chitungwiza Central Hospital are highly active when compared to other studies done in Sub-Saharan Africa. It is also important to create interventions that promote physical activity considering what this population considers as perceived benefits and barriers to physical activity.

## Background

Globally, 38.4 million [33.9–43.8] people are living with HIV [1, 2]. Slightly more than half of the infected people are women and girls (54%);25.6 million (66%) are in Africa; 20.6 million (53%) are in Sub-Saharan Africa (SSA). Those in SSA account for nearly 71% of the total population of people living with HIV (PLWH) worldwide [1]. The incidence rate of HIV decreased by 33% from 2005 to 2013. In 2021, 28.7 million of all people living with HIV were on HAART. A 35% decrease in HIV related deaths occurred worldwide since 2005 to 2013 due to HAART [3]. This has resulted in PLWH living longer making HIV a chronic manageable disease [4]. With this increase in chronicity comes an increase in prevalence and impact of HIV associated complications in this population; many of which can be successfully treated with rehabilitation [4]. Generally, there has been a decrease of HIV prevalence from 26.5% in 1997 to 14.3% in 2021 in Zimbabwe [5]; a 0.31% decrease in incidence rate from 2011 to 2013 [6]. In 2020, Zimbabwe had an adult HIV prevalence of 12.9% (1.23 million adults) [7–9].

Complications of HIV infection include lipodystrophy [10], chronic pain, neurological deficits and musculoskeletal impairments [9]. There is also an increased prevalence of cardiovascular diseases among PLWH. Complications involving the pulmonary system include pulmonary hypertension and lung cancer [11].

Physical activity is a treatment technique employed by rehabilitation personnel in managing complications associated with HIV [12]. Physical activity in HIV positive people has been shown to be beneficial as it improves muscle strength and endurance as well as an increase in the expiratory flow rate [12]. When done regularly, physical activity contributes to an improvement of the quality of life among PLWH. Physical activity has also been shown to improve body fat composition and metabolic profiles as well as cardiovascular fitness in this population [9].

Despite the strong evidence for the benefits of physical activity, in particular regular exercise in people living with HIV, it is not known how well this advice is being taken into action and whether the levels of uptake are sufficient enough to achieve health benefits [13]. Many individuals do not take part in sufficient physical activity due to a (negative perception) low perception of the benefits and high perception of barriers to exercise [14]. Research has mainly focused on the effectiveness of physical activity in particular physical exercise among PLWH and there is limited information on the perception thereof and levels of physical activity among patients living with HIV in Sub-Saharan Africa [15]. Given the strong evidence base for the (positive outcomes) beneficial effects of regular physical exercise for all and particularly for those living with HIV, this research aimed to determine the physical activity levels and the perceived benefits and perceived barriers towards physical activity among adults living with HIV attending the opportunistic infections clinic at Chitungwiza Central Hospital (CCH), Zimbabwe.

## Methods

### Study design and setting

A descriptive quantitative cross-sectional survey was conducted. The study was carried out at CCH; one of the largest referral hospitals in Zimbabwe. It is among the first to offer opportunistic infections (OI) services for PLHIV in Zimbabwe. Here patients receive medical, rehabilitation, maternity and pharmaceutical services. The OI clinic runs daily, 5 days/week for adults living with HIV.

### Participants

Male and female adults aged 18 years and above; accessing antiretroviral therapy at CCH OI clinic at the time of the study for at least one year were recruited into the study. Those too ill to participate and those with other evident comorbid physical conditions/disabilities were excluded.

### Sample size

The calculated sample size was 327 based on sample size calculation for cross-sectional studies. The proportion of PLWH estimated to be involved in physical activity or physical exercise was 30%.

### Instrument

The data collection tool was a questionnaire with three sections. Section A collected socio-demographic information; (age, gender, marital status, and employment status) and clinical data (duration on HAART and CD4 + cell counts). Section B evaluated the participants’ level of physical activity using the International Physical Activity Questionnaire (IPAQ). Section C was the Exercise Benefits/Barriers Scale (EBBS) which assessed the perceived benefits and barriers to physical exercise.

The IPAQ consists of 27 questions grouped into five sections. It assesses different categories of physical activity in areas of the job, transportation, housework, house maintenance, caring for family, recreation, sports and leisure-time as well as time spent sitting. Levels of physical activity are determined in relation to IPAQ scoring protocol. Frequencies and duration of participating in physical activity are also included.

The EBBS comprises the benefits and the barriers to physical activity. The benefits are divided into five sub-scales; (physical performance, life enhancement, psychological outlook, social interaction and preventive health).The barriers are divided into four sub-scales; (exercise milieu, time-expenditure, physical exertion and family discouragement) [14]. There are 29 items for the benefits and 14 items for the barriers. The EBBS has four- Likert type responses, that range from 4 (strongly agree) to 1 (strongly disagree). The higher the benefit scores, the more positively the participant perceives benefits of physical activity. The higher the barriers score, the more negatively the participant perceives physical activity.

The tools were translated to Shona a local vernacular language used in Chitungwiza using the forward-backward translation method. A pilot study was then conducted to validate as well as to test for reliability of the translated version. For reliability the physical activity questionnaire was computed on SPSS and calculation of Cronbach’s alpha yielded a standardized alpha of 0.734. Calculation of Cronbach’s alpha for the Exercise Benefits and Barrier Scale yielded a standardized alpha of 0.912. Other authors [16] found a Cronbach’s alpha coefficients for the total score and the instrument’s subscales to be 0.92, 0.94 and 0.84 respectively. Therefore, these translated versions of the questionnaires were reliable enough to be used for data collection in the main study.

### Data collection

Permission to carry out study at Chitungwiza was granted by the CCH Ethics Committee. Ethical clearance of the study protocol was granted by the Joint Research Ethics Committee for the University of Zimbabwe and Parirenyatwa Group of Hospitals.(JREC 258/15) and the Medical Research Council of Zimbabwe (MRCZ/B/951). Participants willing to participate gave informed written consent. Consecutive sampling was used to recruit participants who met the inclusion criteria until the required number was reached. The data collection tool was self-administered in both Shona and English languages for easy communication with participants. Data was collected whilst patients were waiting to be attended to by the doctor. Data was collected from Monday to Friday over a period of three weeks. The completed questionnaires were collected on the same day. Participants also received advice on the benefits of physical activity after completing the questionnaire.

### Data analysis

Microsoft Excel was used to capture the data. Data was analysed using the Statistical Package for the Social Sciences (SPSS). Continuous data was summarised using means and standard deviations. Categorical data was reported as frequencies and percentages. Single paired t-tests were used to find whether these adults perceived more benefits or barriers to physical exercise. Multiple paired t-tests were used for analysis of sub-scale significant differences. For the benefits scale, ten comparisons were analyzed whereas six comparisons were done for the barrier scale. For the correction of critical p values Bonferroni method using an alpha of 5% was used.

## Results

### Sample characteristics of adults living with HIV

A total of 327 answered the questionnaires. Four were not included for analysis because they did not meet the requirements of the IPAQ scoring protocol for completeness where participants gave “I don’t know” as an answer, walking was more than 16 h and where minimum duration of activity was less than 10 min, leaving 323 questionnaires for analysis.

### Socio-demographic profile of participants

Most of the participants were females (n = 225; 69.7%), mean age was 41.1 ± 11.0 years old. Almost half of the participants were below the age of 40 years (n = 154; 47.8%), 195 (60.6% were married and 171 (53.1%) were employed. Most of the participants were Christians (n = 256; 79.3%).

### Clinical information

The minimum time spent on HAART was one year and the maximum time was 20 years with the mean time on HAART being 4.3 ± 3.2 years. In addition, 202 (62.8%) of the participants had spent more than 5 years on HAART. Current CD4 cell counts of the sample ranged from 6 cells per mm^2^ to 1572 cells per mm^2^ with a mean of 406.6 ± 279.9 cells per mm^2^. Just under half (n = 157; 48.9%) of the participants had CD4 cell counts ≥ 350 cells per mm^2^.

### Physical activity levels of the study sample

**Fig. 1:**
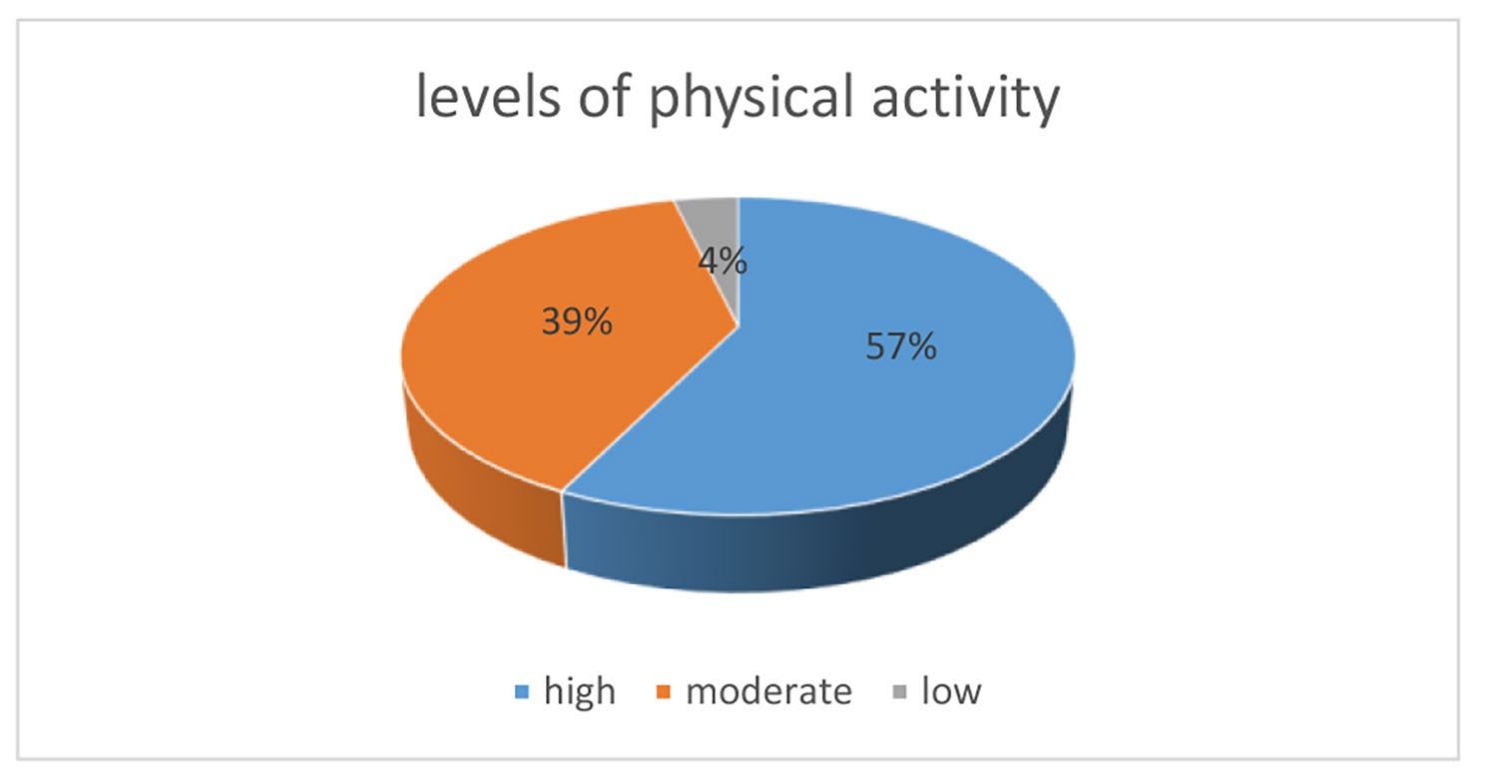
Levels of physical activity

Out of the 323 participants 184 (57.1%) were found to be highly active (3204;IQR = 2139–4441 MET-minutes/week) (Fig. 1). The mean sitting time was 192 ± 93 min per day. The most frequent activity with the most energy expenditure was moderate physical activity which includes household, yard work, moderate activities at work, during leisure time and cycling for transportation with a median value of 1770(IQR = 1060-2620MET-minutes/week). The study participants spent most of their time in sitting with a median value of 1260(IQR = 840–1800 min/week) when compared to other activities. A statistically significant association between levels of physical activity and CD4 cell counts (*p* = 0.035) was found. Participants who had CD4 cell counts that were ≥ 350mm^2^ were found to be highly active. In addition, there was also a statistically significant association between employment status and levels of physical activity (p < 0.05).

### Perceived benefits/barriers to physical exercise

The minimum total score was 32 and the maximum was 116, with a mean of 92.1 ± 13.8. Computed means of each benefit item showed that the participants agreed the most with the item: ‘I will prevent heart attacks by exercising’ (M ± SD = 3.34 ± 0.65), followed by ‘I enjoy exercise (M ± SD = 3.33 ± 0.75) and ‘exercise improves my mental health (feeling happy and emotionally well)’ (M ± SD = 3.33 ± 0.69). ‘My disposition is improved with exercise (mood or general attitude about life)’ (M ± SD = 2.75 ± 0.95), was the least agreed to by the study sample. For all the benefit items, the mean was 3.18 ± 0.48 (Table 1).

**Table 1:** Mean and standard deviation of each exercise benefits scale questionnaire item

PERCEIVED BENEFITS ITEMS	M ± SD
**LIFE ENHANCEMENT SUB-SCALE**	
25. My disposition is improved with exercise (mood or general attitude about life).	2.75 ± 0.95
26. Exercising helps me sleep better at night.	3.26 ± 0.72
29. Exercise helps me decrease fatigue.	3.10 ± 0.81
32. Exercising improves my self-concept (the way in which I think about myself and the image I have of myself).	3.06 ± 0.78
34. Exercising increases my mental alertness (speed of thought or power of concentration).	2.98 ± 0.84
35. Exercise allows me to carry out normal activities without becoming tired.	3.28 ± 0.63
36. Exercise improves the quality of my work	3.10 ± 0.76
41. Exercise improves overall body functioning for me.	3.23 ± 0.68
**PHYSICAL PERFOMANCE SUB-SCALE**	
7. Exercise increases my muscle strength (the ability of a muscle to employ an effort against some resistance e.g. digging)	3.32 ± 0.66
15. Exercising increases my level of physical fitness (the ability to carry out activities without undue tiring).	3.31 ± 0.71
17. My muscle tone (the state of tension inside the muscle that is maintained continuously even at rest or when relaxed and which increases in resistance to stretch) is improved with exercise	3.18 ± 0.70
18. Exercising improves functioning of my heart and circulation of blood in my body.	3.26 ± 0.74
22. Exercise increases my stamina (the ability to maintain prolonged physical or mental effort).	3.23 ± 0.76
23. Exercise improves my flexibility (the ability of joints and muscles to move freely e.g. lifting the arm, bending the body).	3.16 ± 0.77
31. My physical endurance is improved by exercising (the strength to keep going e.g. being able to walk for 30 minutes or more).	3.03 ± 0.83
43. Exercise improves the way my body looks.	3.24 ± 0.72
**PSYCHOLOGICAL OUTLOOK SUB-SCALE**	
1. I enjoy exercise	3.33 ± 0.75
2. Exercise decreases feelings of stress and tension for me.	3.25 ± 0.72
3. Exercise improves my mental health (feeling happy and emotionally well).	3.33 ± 0.69
8. Exercise gives me a sense of personal accomplishment (the successful completion of something).	3.24 ± 0.70
10. Exercising makes me feel relaxed.	3.31 ± 0.69
20. I have improved feelings of wellbeing from exercise (to be healthy, comfortable and happy).	3.15 ± 0.74
**SOCIAL INTERACTION SUB-SCALE**	
11. Exercising lets me have contact with friends and persons I enjoy	3.06 ± 0.87
30. Exercising is a good way for me to meet new people	3.07 ± 0.82
38. Exercise is good entertainment for me.	3.06 ± 0.78
39. Exercising increases my acceptance by others.	2.99 ± 0.84
**PREVENTIVE HEALTH SUB-SCALE**	
5. I will prevent heart attacks by exercising.	3.34 ± 0.65
13. Exercising will keep me from having high blood pressure	3.20 ± 0.75
27. I will live longer if I exercise.	3.23 ± 0.75
**ALL BENEFITS ITEMS**	**3.18 ± 0.48**

The greatest perceived benefit from exercise was psychological outlook (M ± SD = 3.27 ± 0.52), closely followed by preventive health (M ± SD = 3.26 ± 0.56). Multiple paired t-tests for identification of any statistically significant difference between sub-scales showed that psychological outlook was significantly higher (M = 3.27) than all other benefit sub-scales. It was closely followed by preventive health (Table 2).

**Table 2:** Standardized perceived benefit means and standard deviations and differences between sub-scales mean values for multiple comparisons

			BENEFIT SUB-SCALE^				
BENEFIT SUB-SCALE	**MEAN(SD)**		**1**	**2**	**3**	**4**	**5**
1. Life enhancement	3.09(0.53)		--	0.12	0.17*	-0.05	0.16*
2. Physical performance	3.21(0.51)			--	0.05	-0.17*	0.04
3. Psychological outlook	3.27(0.52)				--	-0.22*	-0.01
4. Social interaction	3.05(0.62)					--	0.21*
5. Preventive health	3.26(0.56)						--

Possible scores range from 1 to 4 for all sub-scales. Four represents the highest perception of benefits to physical exercise; ^Values in the cells of these columns are actual differences between sub-scale mean values; * Indicates that the means of the benefits sub-scales that are compared are significantly different, using Bonferroni corrected critical p values for benefits (p < 0.05)

The minimum total score for the barrier items was 14 and the maximum was 56, with a mean of 32.2 ± 6.5. Computed mean for each barrier item showed that the participants agreed the most with the item: ‘it costs too much to exercise’ (M ± SD = 3.00 ± 0.88), followed by, ‘I’m too embarrassed to exercise’, (M ± SD = 2.96 ± 0.88), and ‘my family members do not encourage me to exercise’ (M ± SD = 2.95 ± 0.84). The most disagreed with items were: ‘places for me to exercise are too far away’ (M ± SD = 2.42 ± 0.91), and ‘exercise tires me’ (M ± SD = 2.42 ± 0.95). For all the barrier items, the mean was 2.72 ± 0.47 (Table 3).

**Table 3:** Mean and standard deviation of each exercise barrier scale questionnaire items

PERCEIVED BARIERS ITEMS	M ± SD
**EXERCISE MILIEU SUB-SCALE**	
9. Places for me to exercise are too far away	2.42 ± 0.91
12. I am too embarrassed to exercise.	2.96 ± 0.88
14. It costs too much to exercise.	3.00 ± 0.88
16. Exercise facilities do not have convenient schedules for me.	2.55 ± 0.88
28. I think people in exercise clothes look funny.	2.81 ± 0.90
42. There are too few places for me to exercise.	2.46 ± 0.91
**TIME EXPENDITURE SUB-SCALE**	
4. Exercising takes too much of my time.	2.60 ± 0.83
24. Exercise takes too much time from family relationships	2.61 ± 0.94
37. Exercise takes too much time from my family responsibilities.	2.59 ± 0.93
**PHYSICAL EXERTION SUB-SCALE**	
6. Exercise tires me.	2.42 ± 0.95
19. I am fatigued (state of being very tired) by exercise.	2.65 ± 0.90
40. Exercise is hard work for me.	2.90 ± 0.81
**FAMILY DISCOURAGEMENT SUB-SCALE**	
21. My spouse (wife or husband) does not encourage exercising.	2.89 ± 0.83
33. My family members do not encourage me to exercise.	2.95 ± 0.84
**ALL BARRIER ITEMS**	**2.72 ± 0.47**

Family discouragement (M ± SD = 2.91 ± 0.67) was the most perceived barrier to exercise followed by exercise milieu (M ± SD = 2.70 ± 0.52). Multiple paired t-tests for identification of any statistically significant difference between sub-scales showed that family discouragement was rated significantly higher as compared to the others (Table 4).

**Table 4:** Standardized perceived barrier means and standard deviations and differences between sub-scales mean values for multiple comparisons

		BARRIER SUB-SCALE^			
BARRIER SUB-SCALE	**MEAN(SD)**	**1**	**2**	**3**	**4**
1. Exercise Milieu	2.70(0.52)	--	-0.10	-0.04	0.22*
2. Time Expenditure	2.60(0.67)		--	0.06	0.31*
3. Physical Exertion	2.66(0.62)			--	-0.26*
4. Family Discouragement	2.91(0.67)				--

Possible scores ranged from 1 to 4 for all sub-scales. Four represents the highest perception of barriers to physical exercise; ^Values in the cells of these columns are actual differences between sub-scale mean values; * Indicates that the means of the barriers sub-scales that are compared are significantly different, using Bonferroni corrected critical p values for barriers (p < 0.001)

No further differences were noted between exercise milieu, time expenditure and physical exertion. Possible scores ranged from 1 to 4 for all sub-scales. No statistically significant association was reported between the perceived barriers and the level of physical activity. A statistically significant association was reported between the perceived barriers and age (p <0.001) as well as with employment status (p = 0.006). No other statistically significant association was noted among the other personal characteristics and perceived barriers to physical exercise.

## Discussion

A little more than half of the sample (57.0%) in this study were highly active. This may be because the majority are women who are expected to maintain their roles in the home. Most of the PLWH are within the working age group hence may still be active at work and they are encouraged to be physically active so that they may live longer [13]. In addition, Littlewood et al., [17] suggested that HIV diagnosis results in a positive health behaviour change; in this case, regular physical activity participation. The finding that the activity with the most energy expenditure was moderate physical activity (yardwork, inside chores etc.); suggests that the participants might not be aware of the need to vigorously exercise for the benefit of their health. Moderate physical activity is being done to run house and other day to day activities necessary at home. Participants spent most of their time sitting which is a cause for concern as it is a risk factor for cardiovascular disease among people living with HIV [18]. On the other hand, considering that the majority were highly active may suggests that the time spent sitting was for resting and energy conservation. Furthermore, the current economic challenges in Zimbabwe results in people being highly active doing menial jobs to aid their financial needs. The standard practice in the ART programme is that PLWH are encouraged, by counsellors, to engage in healthy habits including regular exercise.

The general levels of perceived benefits to physical exercise showed that adults living with HIV ‘strongly agreed ‘or ‘agreed’ with most of the benefit items. All the five sub-scales of the benefits demonstrated standardized means of > 3 thus representing ‘true’ agreement that all these benefit items had factors that the participants viewed as benefits from regular physical exercise participation. The study results revealed that psychological outlook was the most perceived benefit to physical exercise among the sub-scales of the benefit components, which indicated that participants perceived physical exercise as a way of reducing depression as well as improving their wellbeing in general. Furthermore, getting an HIV positive diagnosis is stressful in nature hence the need for exercises which is deemed beneficial in this psychological domain of benefits.

It is encouraging that these adults living with HIV perceived psychological components as benefits to physical exercise as this supports the evidence that psychological wellbeing is improved by increasing the levels of physical activity [18]. Furthermore, this finding was also in agreement with the evidence that physical activity participation results in better mental health [19]. Participants perceived relatively fewer benefits from physical exercise in relation to life enhancement and social interaction. The reason might be that after being diagnosed of HIV, the participants might have lost a sense of belonging, hence physical training for them became a source of interaction with like people whom they could openly interact with without Stigma.

Participants fairly agreed with many of the barrier items indicating that most of these items were an actual presentation of the barriers to physical exercise participation among the participants. Participants agreed the most with, ‘it costs too much to exercise’ with a mean of 3.00 ± 0.88. Therefore, these participants were less likely to exercise due to the perception that they had to go somewhere and pay for the services to be physically active. PLWH felt that family discouragement was the most perceived barrier to physical exercise among all the barrier sub-scales. This is of concern as the results of this present study indicates that these participants were more likely not to engage in physical exercise due to the lack of support from their family members. Another possible explanation could be social cultural beliefs or systems of sick-role, care giving, interdependence and over-protective care givers which promote less activity in PLWH as highlighted among stroke patients [20].

Participants had a limited perception of time expenditure as a barrier to exercise. This limited perception of time expenditure as a barrier to physical exercise is positive as this indicates potential time to engage in physical exercise among the study participants [14]. The study results showed that a statistically significant association existed between the perceived barriers and age (p < 0.05) which suggest that those who are older were less likely to engage in physical exercise. Furthermore, the finding that a statistically significant association existed between the perceived benefits of exercise and employment (p < 0.05), suggests that employed individuals are more likely to exercise as they perceive less barriers than unemployed individuals. In addition, it is socially accepted in the Zimbabwean context that vigorous activity is associated with the young age, moreover employment opportunities favour young adults than those who would have grown up and working towards retirement.

A statistically significant association existed between levels of physical activity and CD4 cell counts (*p* = 0.035). Evidence supports that regular physical activity participation results in an increase in CD4 + cell counts thereby improving the immune system, however, this was not tested in this study [21]. A statistically significant association existed between employment status and levels of physical activity (p < 0.05). The possible explanation could be that employed people are more likely to be physically active than unemployed as they have to travel to work and maybe do physical activity as part of their daily routine for long hours, while the unemployed have to choose the time to do activities and rest as their targets are not salary based. Moreover, the employment opportunities are very low, considering the economic challenges, thus most of the available jobs are labour related not office related, and some people maybe self-employed as working directors moulding bricks or doing farming activities.

This present study had several limitations. Comparisons with other studies was difficult as different questionnaires were used to measure the levels of physical activity. The study participants were from one hospital in Chitungwiza thus limiting the generalisation of these results to other settings like rural areas. The questionnaire used to measure physical activity levels was also a limitation as it subjectively measures physical activity and there is a tendency of participants to over-estimate or under-estimate the time, they spend doing a particular activity. In addition, the cross-sectional design could not establish cause and effect.

## Conclusion

It is clearly evident that the majority of the participants in this study were highly active. It is of importance to promote and encourage an active life among PLWH as they are burdened by the disease and the side effects of HAART and the effects the virus poses on their health. It is also important to create interventions that promote physical activity considering what this population considers as perceived benefits and barriers to exercise. It is essential for rehabilitation, health education and promotion personnel to design programmes on the importance of physical exercise to increase physical activity levels among adults living with HIV. Rehabilitation personnel should also design and implement structured exercise programmes in hospitals for HIV infected people. Further research to investigate the association between physical activity levels and anthropometric measurements like height, weight and BMI in our setting may be necessary. The actual facilitators and barriers to physical exercise must also be investigated so as to develop exercise programs tailored specifically for this population.

## Data Availability

The datasets used and/or analysed during the current study are available from the corresponding author on reasonable request.

## Abbreviations

HIV: Human Immunodeficiency Virus.
AIDS: Acquired Immunodeficiency Syndrome
UNSAIDS: Joints United Nations Programme on HIV/AIDS
OI: Opportunistic Infections
PLWH: People Living with HIV
WHO: World Health Organisation
HAART: Highly Active Antiretroviral Therapy
ART: Antiretroviral Therapy
CCH: Chitungwiza Central Hospital
EBBS: Exercise Benefits/Barrier Scale
IPAQ: International Physical Activity Questionnaire
JREC: Joint University of Zimbabwe, College of Health Sciences and Parirenyatwa Group of Hospitals Ethics Committee
MRCZ: Medical Research Council of Zimbabwe

## References

[1] Global, HIV &AIDS statistics. 2022. https://www.unaids.org/en/resources/fact-sheet. Accessed 24 September 2022.

[2] World Health Statistics., 2021. https://www.who.int/data/gho/publications/world-health-statistics. Accessed 03 March 2022.

[3] UNAIDS. 2014. Regional statistics. https://unaids-test.unaids.org/sites/default/files/unaids/contentassets/documents/unaidspublication/2014/UNAIDS_Gap_report_en.pdf. Accessed 17 April 2019.

[4] Pullen SD, Chigbo NN, Nwigwe EC, Chukwuka CJ, Amah CC, Idu SC. Physiotherapy intervention as a complementary treatment for people living with HIV/AIDS. HIV AIDS (Auckl). 2014 Jun 2;6:99–107. doi: 10.2147/HIV.S62121. PMID: 24936132; PMCID: PMC4047833. 10.2147/HIV.S62121 PMC4047833 24936132

[5] Fact Sheet – HIV Decline in Zimbabwe. Positive Behaviour change makes a difference. 2022. https://zimbabwe.unfpa.org/sites/default/files/pub-pdf/FACTSheetHIVDeclineinZimbabweFinal.pdf. Accessed 26 November 2022.

[6] Zimbabwe AIDS, Response Progress Report. *Global AIDS Response Country Progress Report Zimbabwe* 2014. https://www.unaids.org/sites/default/files/country/documents/ZWE_narrative_report_2014.pdf. Accessed 17 April 2017

[7] Zimbabwe Population-based HIV Impact Assessment (ZIMPHIA). 2020. https://phia.icap.columbia.edu/wp-content/uploads/2020/11/ZIMPHIA-2020-Summary-Sheet_Web.pdf. Accessed 14 August 2021.

[8] UNAIDS Data 2021. Global & Regional Data. https://www.unaids.org/sites/default/files/media_asset/JC3032_AIDS_Data_book_2021_En.pdf. Accessed 13 October 2022.

[9] UNFPA., 2022. https://zimbabwe.unfpa.org/en/topics/hiv-aids-4. Accessed 26 November 2022.

[10] Singhania R, Kotler D. 2011. Lipodystrophy in HIV patients: its challenges and management approaches. *HIV/AIDS* - *Research and Palliative Care*, 3, pp.135–143. Available at: http://www.pubmedcentral.nih.gov/articlerender.fcgi?artid=3257972&tool=pmcentrez&rendertype=abstract. 10.2147/HIV.S14562 PMC3257972 22267946

[11] Chu C, Selwyn PA (2011). Complications of HIV infection: a systems-based approach. Am Family Phys.

[12] Nixon S, O’Brian K, Glazier R, Tyan AM. Aerobic exercise interventions for adults living with HIV / AIDS (Review). 2009 (1).

[13] Fillipas S, Bowtell-Harris CA, Oldmeadow LB, Cicuttini F, Holland AE, Cherry CL (2008). Physical activity uptake in patients with HIV: who does how much?. International J STD AIDS.

[14] Lovell GP, Ansari WEL, Parker JK (2010). Perceived exercise benefits and barriers of non-exercising female university students in the United Kingdom. Int J Environ Res Public Health.

[15] Frantz JM, Murenzi A (2013). The physical activity levels among people living with human immunodeficiency virus/acquired immunodeficiency syndrome receiving high active antiretroviral therapy in Rwanda. J Social Aspects HIV/AIDS.

[16] Leila Amiri Farahani, Soroor Parvizy, Eesa Mohammadi, Mohsen Asadi-Lari, Anoshiravan Kazemnejad, Seyede Batool Hasanpoor-Azgahdy, and Ziba Taghizadeh. The psychometric properties of exercise benefits/barriers scale among women. Electron Physician. 2017 Jul; 9(7): 4780–5. 10.19082/4780 PMC5586993 28894535

[17] Littlewood R, Vanable P, Michael P (2011). The Association of Benefit finding to Psychosocial and Health Behavior Adaptation among HIV + Men and Women. J Behav Med.

[18] Fillipas S, Cicuttini FM, Holland AE, Cherry CL (2013). Physical activity participation and Cardiovascular Fitness in People living with human immunodeficiency virus: a one- year longitudinal study. J AIDS Clin Res.

[19] Salmon P (2001). Effects of physical exercise on anxiety, depression, and sensitivity to stress. Clin Psychol Rev.

[20] Mudzi W. Impact of caregiver education on stroke survivors and their caregivers. University of Witwatersrand; 2010. PhD Thesis.

[21] Luz E, Sampaio E, Souza R (2011). A Randomized Clinical Trial to evaluate the impact of regular physical activity on the quality of life, body morphology and metabolic parameters of patients with AIDS in Salvador, Brazil. J Acquir Immunedeficiency Syndrome.

